# Achieving universal eye health coverage: planning and human resource lessons from trachoma

**Published:** 2018-07-31

**Authors:** Paul Courtright, Susan Lewallen, Tim Jesudason

**Affiliations:** 1 Co-Founder: Kilimanjaro Centre for Community Ophthalmology & Trachoma Technical Lead (Trust & DFID) Sightsavers.; 2 Co-founder Kilimanjaro Centre for Community Ophthalmology.; 3 Communications Specialist: International Coalition for Trachoma Control London, UK.

**Teamwork has made a crucial difference to the success of trachoma elimination programmes. However, more trained supervisors are needed.**

**Figure:**
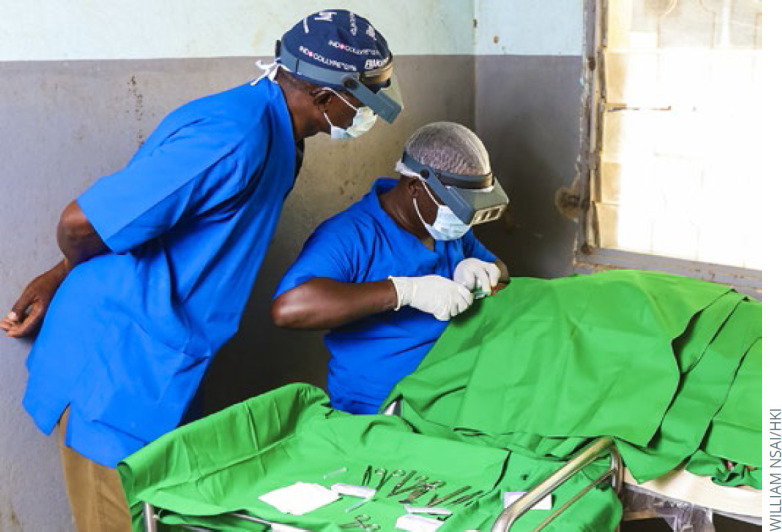
A senior ophthalmic technician, left, supervises a trichiasis surgeon during a mobile surgery campaign in June 2017. CAMEROON

In October 2018, the World Health Organization (WHO) will publish its World Report on Vision. The report will provide a strategic path for the achievement of universal eye health coverage (UEHC). As the world's leading infectious cause of blindness, lessons from the global trachoma elimination programme will support conversations on the achievement of UEHC.

Significant progress has been made to eliminate trachoma through the implementation of the WHO-endorsed SAFE strategy, including elimination in seven countries. However, in order to sustain the gains made, trachoma services must be embedded into routine public services. This will allow individual cases to be managed even as countries reach elimination thresholds. Mobilising the full range of health workers involved in the implementation of the SAFE strategy will be important to maintain high quality case management and to strengthen health systems.

Lessons learned from programmatic efforts to manage trachomatous trichiasis (TT) in sub-Saharan Africa inform us of three ways to strengthen the principles of UEHC, which will be relevant to other eye health issues detailed in the report. These include the importance of district micro-planning; effectively using limited human resources, and adopting pro-active supportive supervision to improve the quality of care.

1. **Carrying out district–level micro-planning of trichiasis services** ensures that all people with trichiasis can be accurately identified and offered services. Micro-planning is a process where maps are used to divide the district into smaller manageable units and (a) decide which communities will be linked to each specific outreach site, (b) train case finders in these communities to identify suspected trichiasis cases, and (c) plan outreach based upon the findings from the case finding. Case finding and outreach will not be appropriate for all eye care services; however, we must recognise the value of service delivery mapping in order to strategically plan how to reach patients.
2. **Strategies to effectively use limited human resources are crucial in resource-poor settings**. Training ophthalmic nurses and ophthalmic clinical officers to manage TT surgeries is common in many trachoma endemic countries, including Kenya, Tanzania and Chad. Research shows that these personnel can be highly productive and capable TT surgeons,^1^ making them essential to deliver TT surgical services at the scale needed to achieve elimination. In Ethiopia, the large backlog of trichiasis patients requiring surgical services and a lack of human resources has resulted in ‘task-shifting’ – the training of general health workers to provide eye care services, including TT surgery. However, due to external factors such as poor mobile phone coverage and weak transport at many hard-to-reach health facilities, some surgeons do not remain in these settings very long and attrition levels are high.
  In order to achieve and sustain elimination, trachoma programmes need to employ and support people with the right set of skills to complete the wide range of tasks needed for efficient service delivery. Strong trichiasis surgical teams include workers who counsel patients, assist with surgery, sterilise instruments, and coordinate activities. This has enabled programmes to significantly increase both their effectiveness and efficiency. Strengthening general eye care services requires a similar approach to team development.
3. **The adoption of pro-active supportive supervision is critical to improve the quality of care.** Evidence shows that TT surgeons provided with supportive supervision focused on the quality of surgery and the efficiency of service delivery, and improved all aspects of patient care.^1^ Training in supportive supervision, and the adoption and use of supervision checklists, have helped clarify the role and activities of supervisors. The shortage of trained supervisors continues to vex programmes, however. Training and empowering supervisors is a key activity for all eye care programmes to ensure the delivery of quality care to all.

Momentum for UEHC is growing. In April 2018, 53 heads of state across Asia, Africa and the Pacific re-committed to the elimination of trachoma and bringing vision to everyone, everywhere. The World Report on Vision and lessons from trachoma will be an important resource to enable ministries of health and partners to bring a wide range of experience and evidence to support equitable access and quality eye health care for all.
